# Supplementary material for: An ankyrin-repeat and WRKY-domain-containing immune receptor confers stripe rust resistance in wheat
Source: Nat Commun. 2020 Mar 13;11:1353. doi: 10.1038/s41467-020-15139-6 (PMC7070047; doi:10.1038/s41467-020-15139-6)
Supplement: Supplementary file 1 — Supplementary Information [file 41467_2020_15139_MOESM1_ESM.pdf]

An ankyrin-repeat and WRKY-domain-containing immune receptor confers stripe rust resistance in wheat

Wang *et al*.

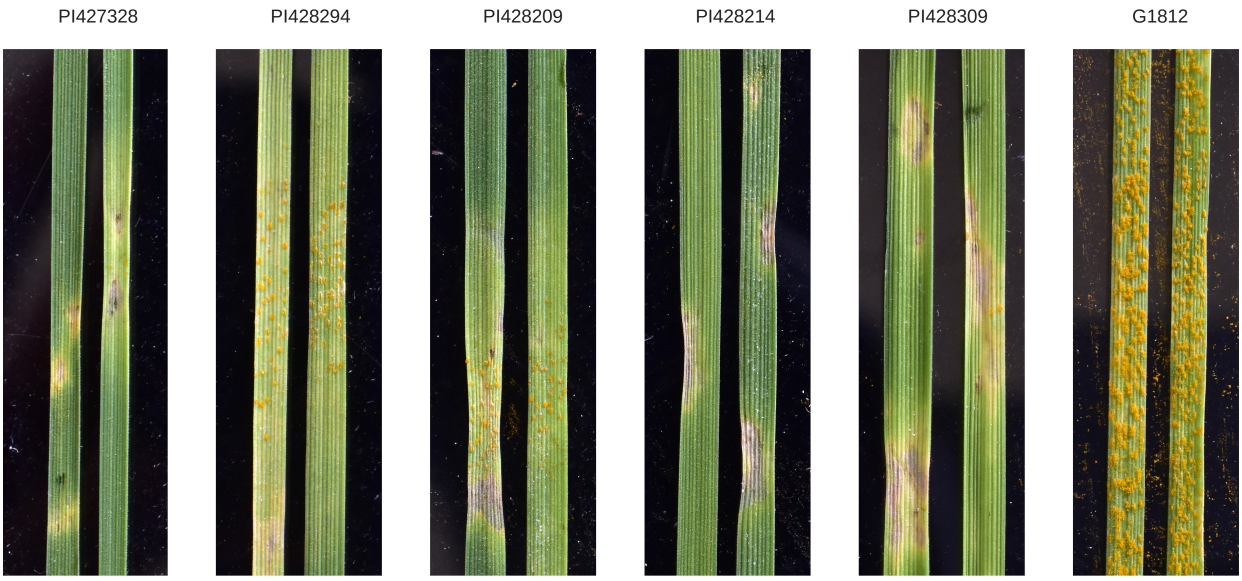

Supplementary Fig. 1 | Five *T. urartu* accessions, including PI428309, were resistant to *Pst* CYR33. Ten-day-old seedlings of PI428309, G1812 and various other *T. urartu* accessions were infected with *Pst* CYR33. PI428309 and four other *T. urartu* accessions were resistant to *Pst* CYR33; G1812 was susceptible. The leaves were detached and photographed at 14 dpi.

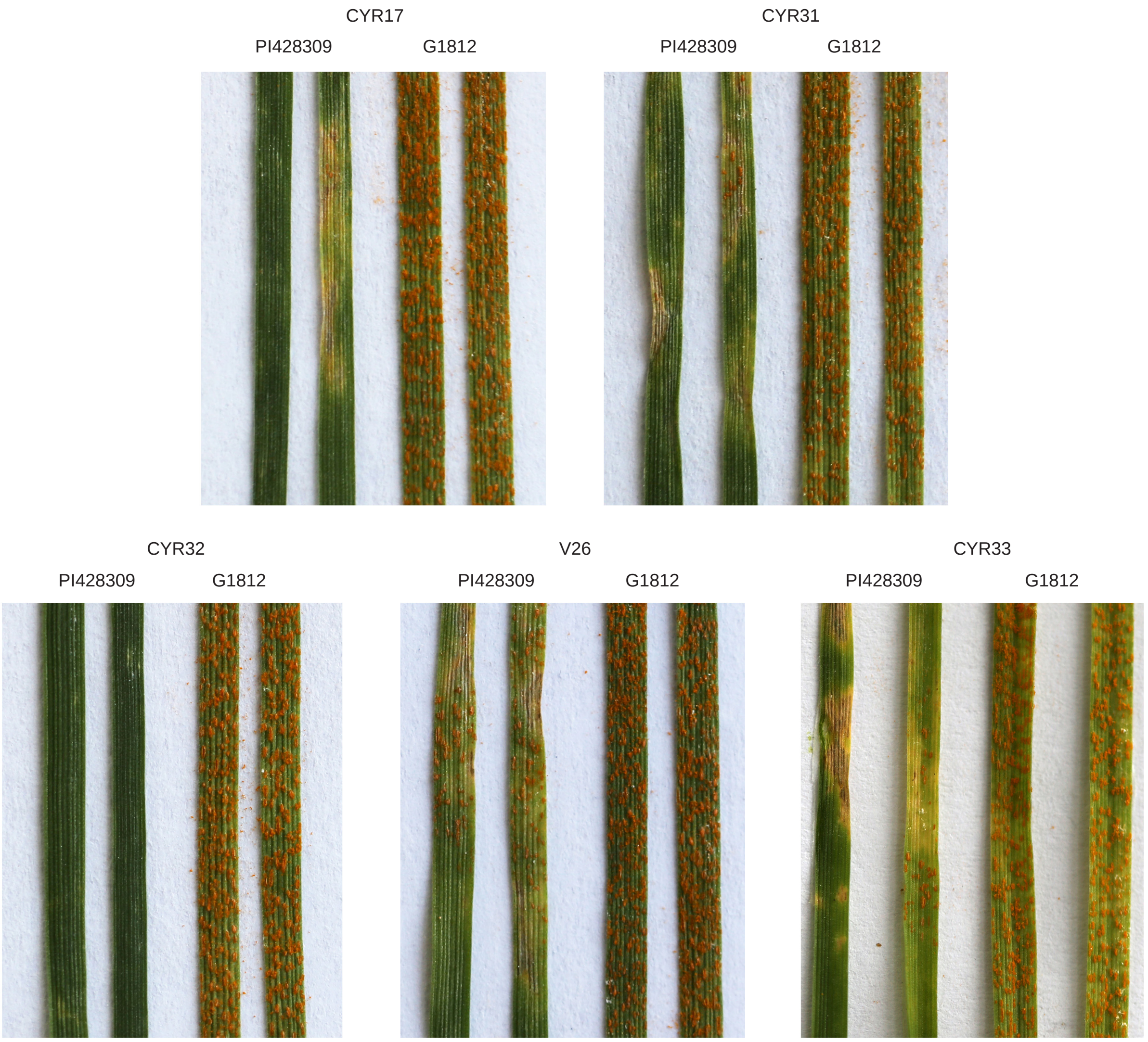

Supplementary Fig. 2 | PI428309 was resistant to five races of *Pst* stripe rust. Infection phenotypes produced by five *Pst* races on PI428309 and G1812. Ten-day-old PI428309 and G1812 seedlings were infected with stripe rust. The leaves were detached and photographed at 14 dpi. PI428309 showed resistance to *Pst* races of CYR33, CYR32, CYR31, CYR17 and V26, while G1812 showed susceptibility.

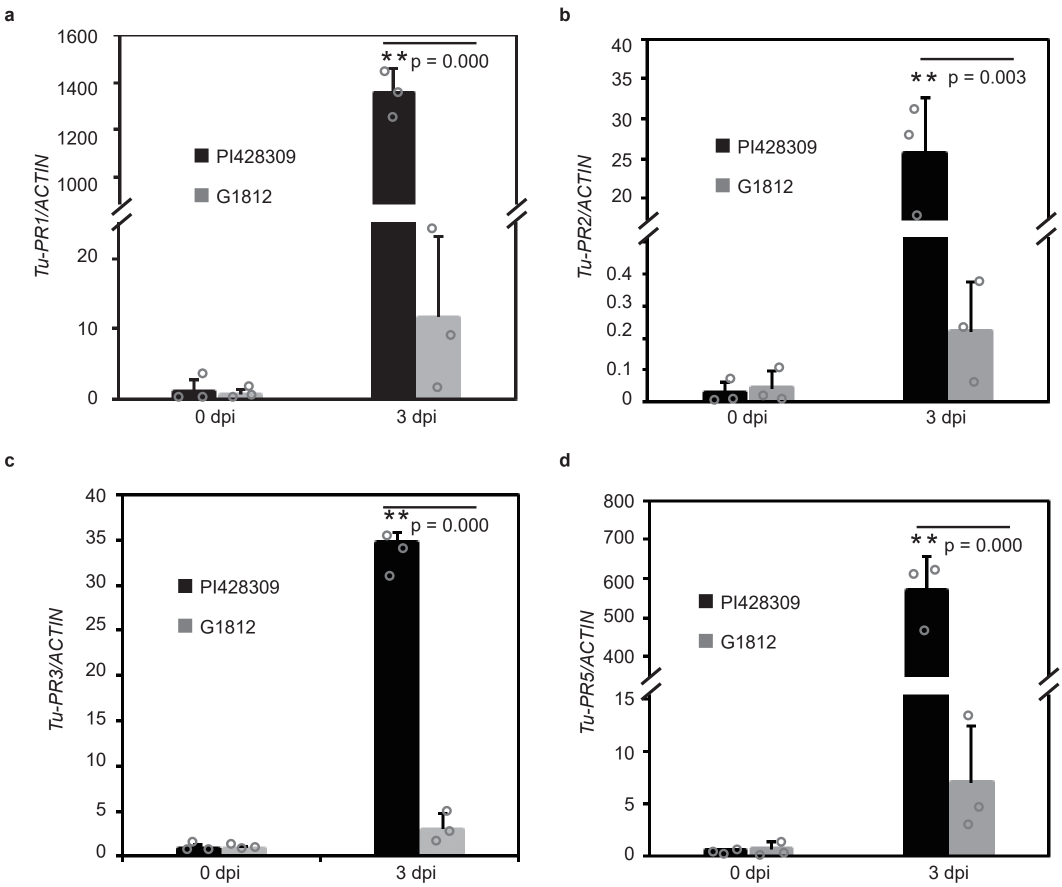

Supplementary Fig. 3 | Relative expression of *PR* genes. Increased relative expression of *TuPR1* **(a)**, *TuPR2* **(b)**, *TuPR3* **(c)** and *TuPR5* **(d)** in PI428309 and G1812. Relative transcript levels of pathogenesis-related genes were examined by quantitative reverse transcription PCR (qRT-PCR). Leaves were collected at 0 and 3 days after infected with stripe rust *Pst* CYR33. Results represent the means ± SD from at least three independent biological samples. Two asterisks indicate statistically significant difference ($P < 0.01$, One-Way ANOVA).

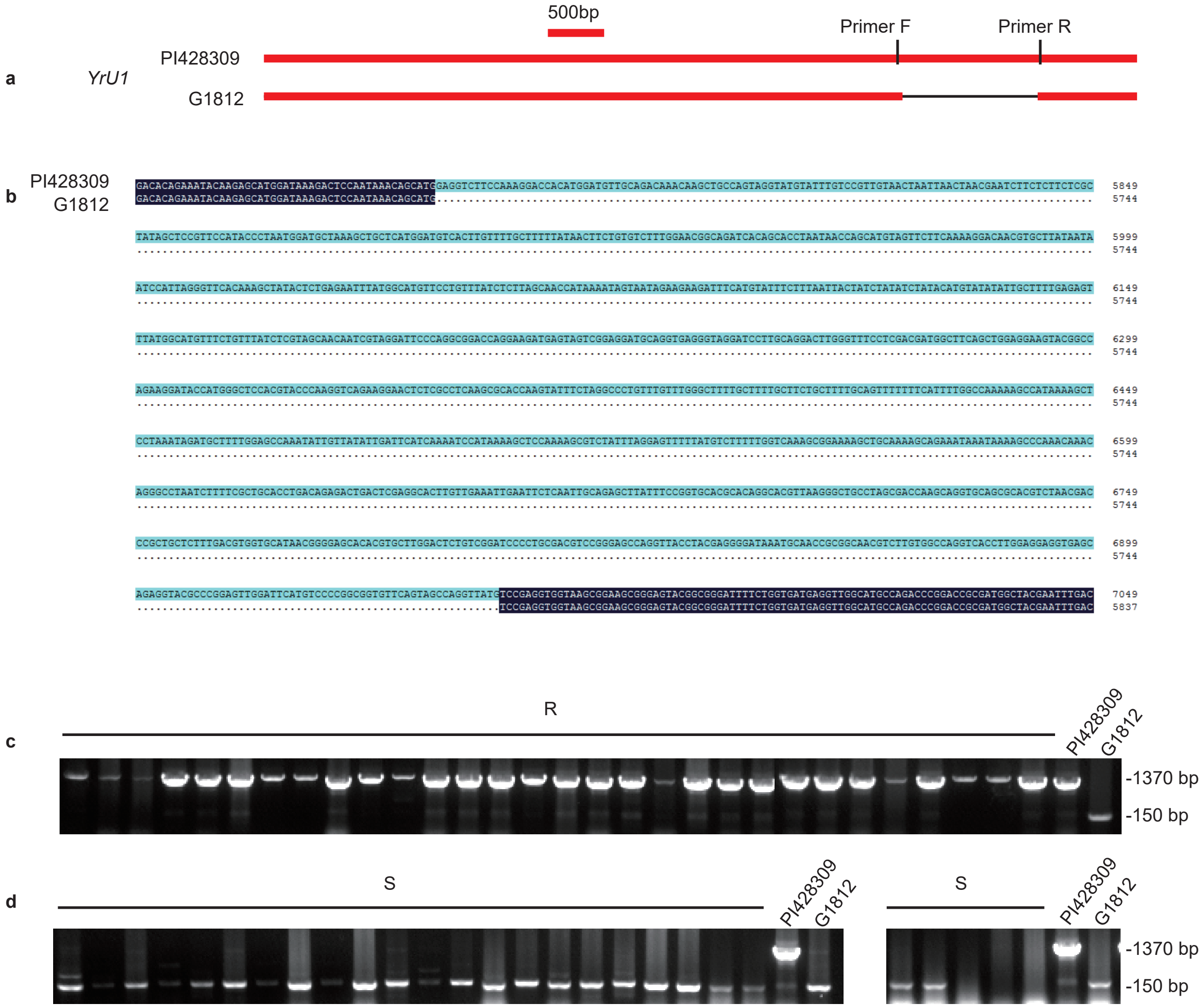

Supplementary Fig. 4 | Sequence analysis of *YrU1* gene in different *T. urartu* accessions. a, Sequence comparison between PI428309 and G1812 of *YrU1*. FP and RP are the primers for the specific marker YrU1-M, which is the sequence that differs between PI428309 and G1812. **b,** Sequence alignment of the *YrU1* gene sequences from PI428309 and G1812. **c,** Genomic fragments of different *T. urartu* accessions that showed resistance to *Pst* CYR33 were amplified with YrU1-M. **d,** Genomic fragments of different *T. urartu* accessions that showed susceptibility to *Pst* CYR33 were amplified with YrU1-M.

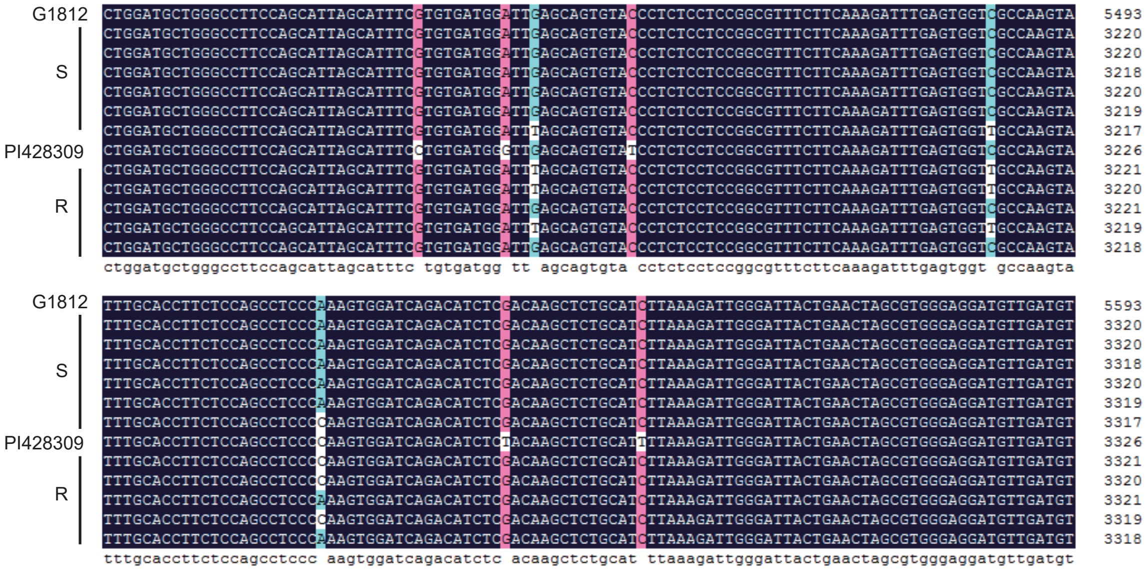

Supplementary Fig. 5 | Sequence alignment of *CG2* gene in different *T. urartu* accessions. There were no significant differences in the sequences of *CG2* between susceptible and resistant *T. urartu* accessions except for some SNPs and short DNA fragments. S, *T. urartu* accessions susceptible to *Pst* CYR33; R, *T. urartu* accessions resistant to *Pst* CYR33.

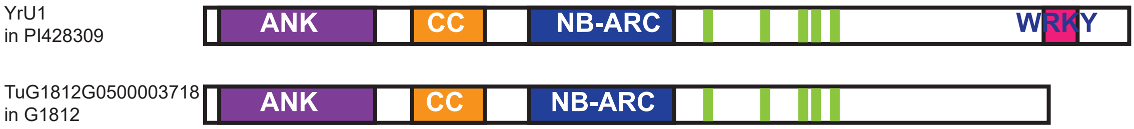

Supplementary Fig. 6 | Structural prediction of candidate *Pst*-resistance proteins in PI428309 and G1812. Structural prediction of candidate *Pst*-resistance proteins using SMART, CD-search and LRRsearch showed that YrU1 is a CC-NBS-LRR (NLR) protein with an additional N-terminal ankyrin-repeat (ANK) domain and C-terminal WRKY domain. TuG1812G0500003718 is a CC-NBS-LRR (NLR) protein with an additional N-terminal ANK domain.

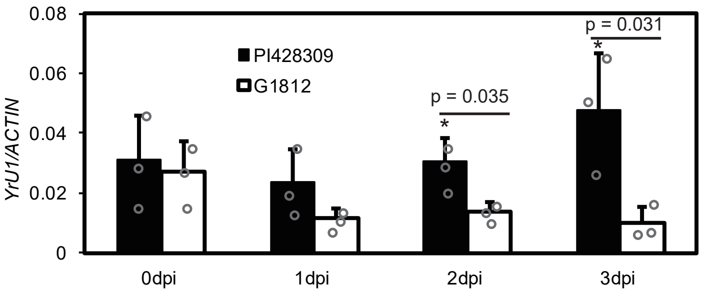

Supplementary Fig. 7 | Transcript levels of *YrU1* in PI428309 and G1812 inoculated with *Pst* CYR33. The expression of *YrU1* was only slightly affected by infection in PI428309. *YrU1* transcript levels were examined by quantitative reverse transcription PCR (qRT-PCR). Leaves were collected at 0, 1, 2, 3 and 4 dpi. Results represent the means ± SD from at least three independent biological samples. Asterisks indicates statistically significant difference ($P < 0.05$, One-Way ANOVA).

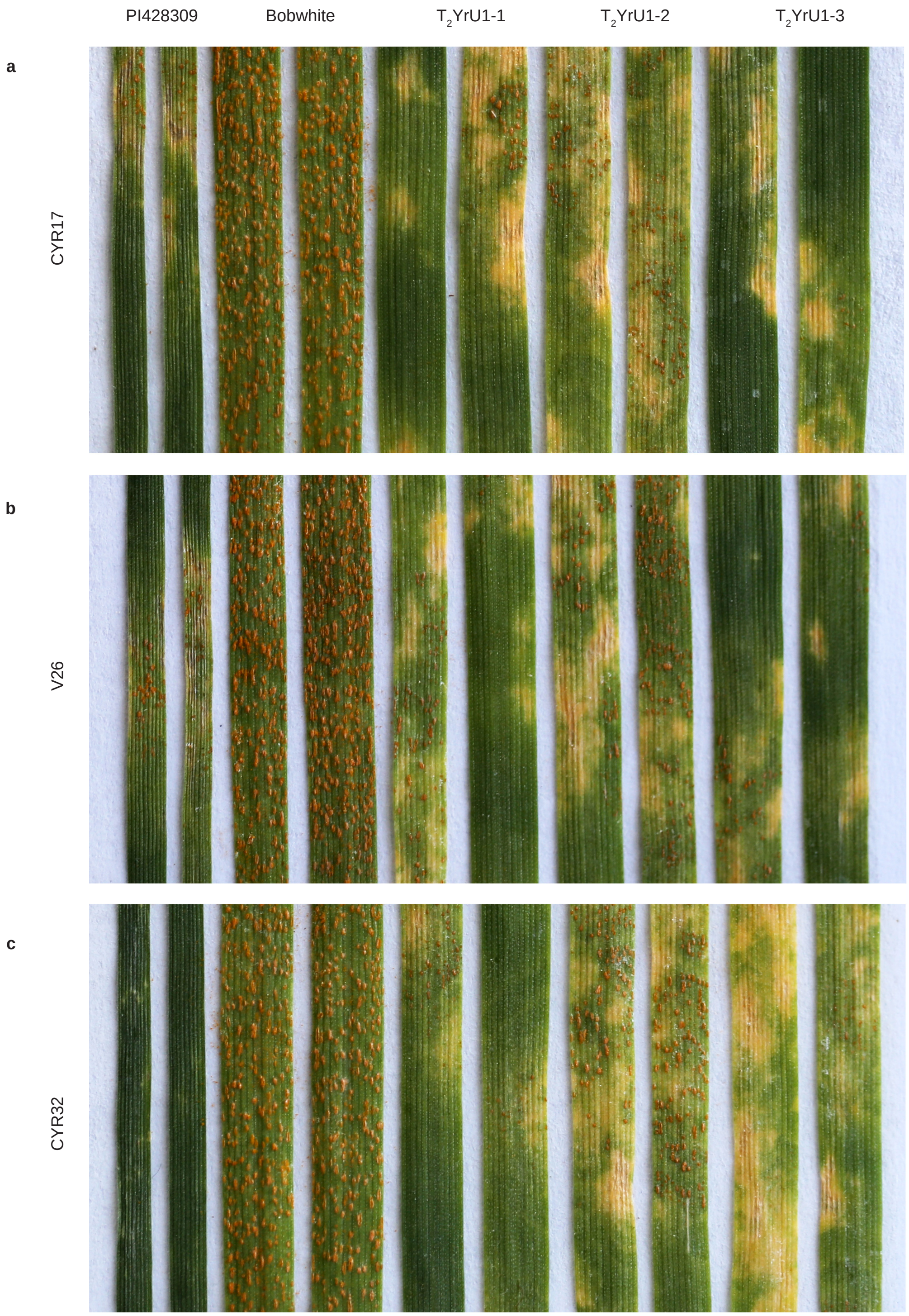

Supplementary Fig. 8 | Results of *YrU1* T_2 transgenic plant inoculation with *Pst* races CYR17, V26 and CYR32. (**a**) CYR17. (**b**) V26. (**c**) CYR32. T_2YrU1-1, T_2YrU1-2 and T_2YrU1-3 were derived from three independent lines (Bobwhite background). All T_2 transgenic plants were resistant to all three *Pst* races.

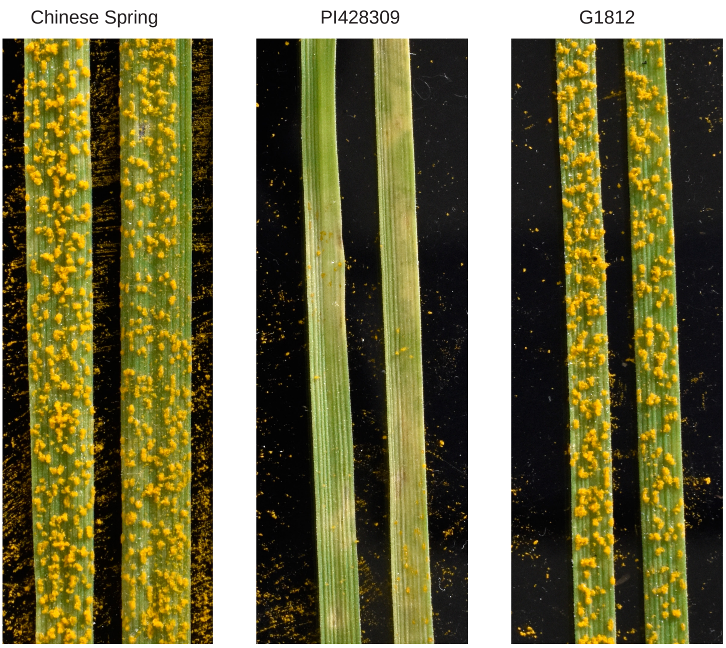

Supplementary Fig. 9 | *Triticum aestivum* wheat cultivar Chinese Spring is susceptible to stripe rust *Pst* CYR33. Ten-day-old Chinese Spring, PI428309 and G1812 seedlings were infected with stripe rust. The leaves were detached and photographed at 14 dpi.

a

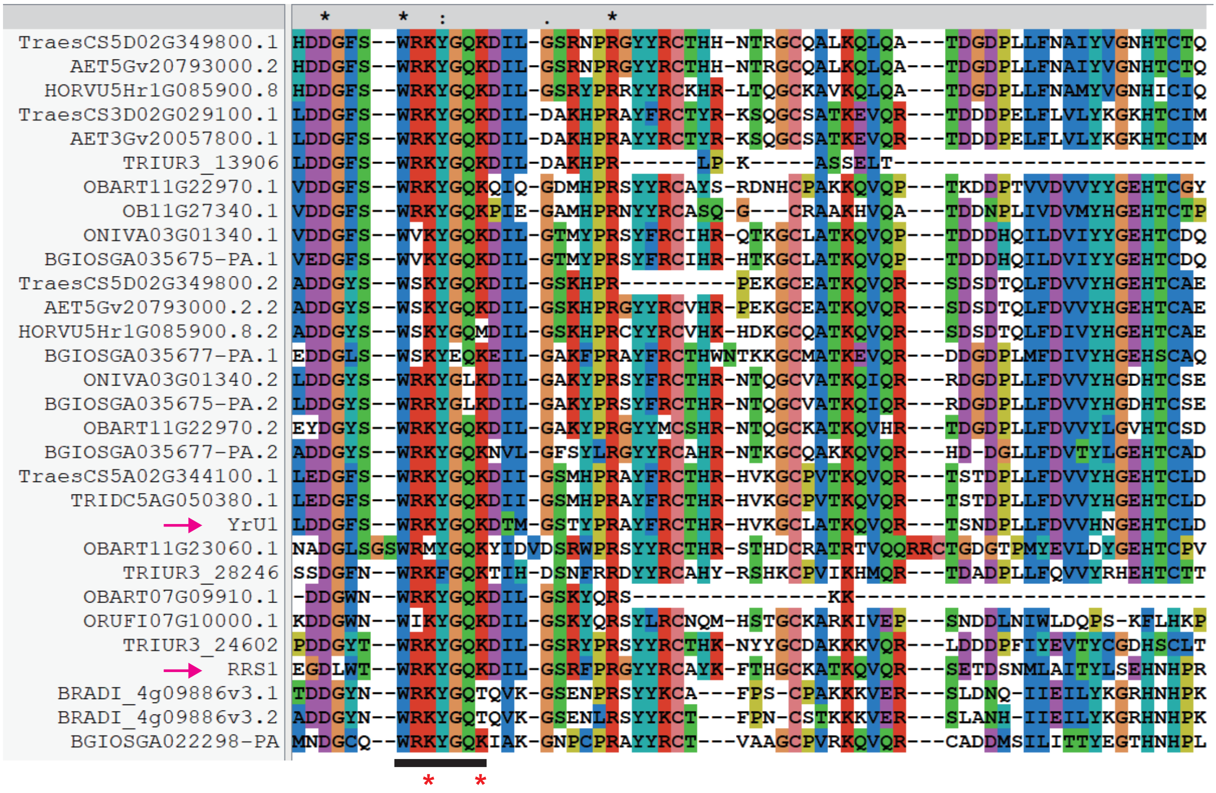

b

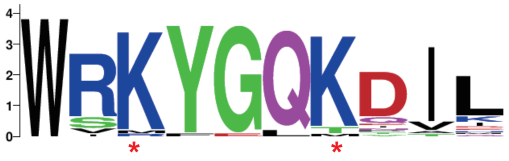

Supplementary Fig. 10 | Sequence alignment of WRKY domains from the 17 NLR-WRKYs and RRS1. a, Sequence alignment of WRKY domains from the 17 NLR-WRKYs and RRS1 was performed with ClustalX 2.1. YrU1 and RRS1 are indicated by red arrows and the WRKY heptad domain by a black line. **b,** WebLogo (http://weblogo.berkeley.edu/logo.cgi) diagram showing that the WRKY heptad domains are relatively conserved.

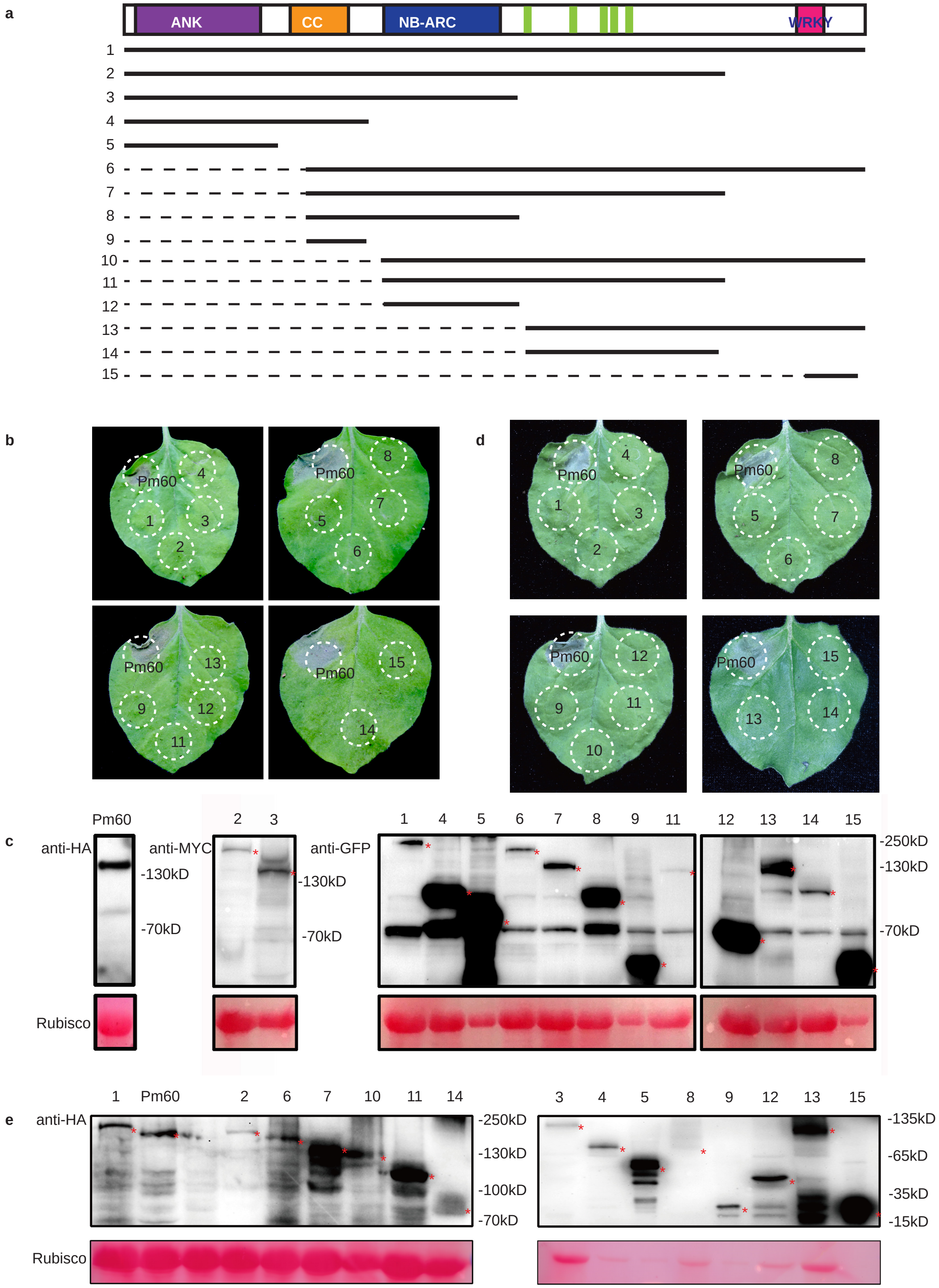

Supplementary Fig. 11 | Cell death was not observed after transient expression of YrU1 in *N. benthamiana*. a, Structure of the YrU1 protein. 1 is full-length YrU1; 2-15 are different domains of YrU1. **b, d** Full-length YrU1 and different domains with (**b**) N-terminal tag or (**d**) C-terminal HA tag were transiently expressed in *N. benthamiana* and then cell death was detected at 48 hpi. Pm60 was used as a positive control. **c, e** Total protein was extracted from leaves of *N. benthamiana* at 48 hpi. The total protein was separated by SDS-PAGE gel and subjected to immunoblot analysis with anti-MYC, anti-GFP and anti-HA antibody, (**c**) N-terminal tag, (**e**) C-terminal tag. Pm60 was used as a positive control. Ponceau S staining of Rubisco is shown below as a loading control. Numbers represent different domains of YrU1 and correspond to the numbers in **a**; red asterisks indicate the target proteins.

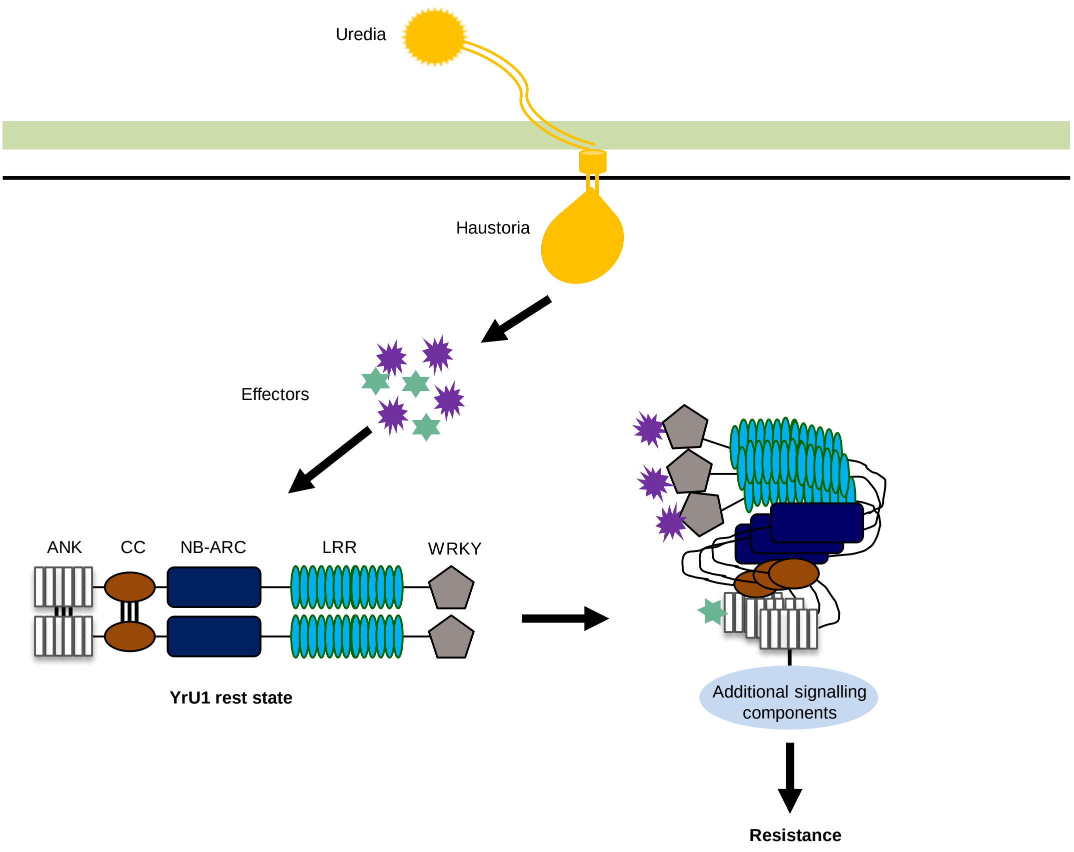

Supplementary Fig. 12 | Working model of YrU1 function. In this model, YrU1 is in the rest state in the absence of pathogen. When stripe rust pathogen is present, the effector binds to the WRKY domain, resulting in conformation changes and oligomerisation of YrU1, which leads to activation of disease resistance. The ANK domain may recruit additional components to activate downstream signalling, or it may act as a decoy for effector binding.

Supplementary Table 1 Segregation ratio of resistance to *Pst* CYR33 in F_2 populations of the cross between PI428309 and G1812

Generation	Reaction types		Total plants	Ratio of Segregation	$\chi2$, P
	R	S			
PI428309	40		40		
G1812		40	40		
PI428309/G1812 F_1	100		100		
PI428309/G1812 F_2	8872	3304	11906	3:1	1.481, 0.224

Supplementary Table 2 Markers used for map-based cloning were designed using wheat expressed sequence tags (ESTs) and scaffolds of G1812

Marker	Scaffold	Deletion-line bin	Type
SCF1	scaffold11003	5AL12-0.35-0.57	SSR
SCF2	scaffold39811	5AL10-0.57-0.78	SSR
SCF3	scaffold60739	5AL10-0.57-0.78	SSR
SCF4	scaffold10881	5AL10-0.57-0.78	SSR
SCF5	scaffold69864	5AL10-0.57-0.78	SSR
SCF6	scaffold49185	5AL10-0.57-0.78	SSR
SCF19	scaffold9979	5AL10-0.57-0.78	SSR
SCF20	scaffold49434	5AL10-0.57-0.78	SSR
SCF12	scaffold68558	5AL10-0.57-0.78	SSR
SCF13	scaffold21752	5AL10-0.57-0.78	SSR
SCF14	scaffold40362	5AL10-0.57-0.78	SSR
SCF15	scaffold7989	5AL10-0.57-0.78	SSR
SCF16	scaffold10281	5AL10-0.57-0.78	SSR
SCF17	scaffold9935	5AL10-0.57-0.78	SSR
SCF18	scaffold10952	5AL10-0.57-0.78	SSR

Supplementary Table 3 Markers used for map-based cloning were designed using *Brachypodium distachyon* genes collinear to the resistance locus and the scaffolds of G1812

**Marker**	**Scaffolds of G1812**	**Genes of *Brachypodium distachyon***	**Type**
SCF10	Scaffold24389	BRADI1G36979<br>BRADI1G36990<br>BRADI1G37010	SSR
SCF11	Scaffold50168	BRADI1G36977	SSR
SCF21	Scaffold45920	BRADI1G36978<br>BRADI1G37090	SSR
SCF22	Scaffold12794	BRADI1G37070	SSR

Supplementary Table 4 Different *Triticum urartu* accessions detected by the *YrU1* gene specific marker.

Reaction types to CYR33	No.	*YrU1*	Country of collection	Accession number
R	64	+	Armenia	PI428257
			Iran	PI662239, PI662238
			Jordan	PI662264, PI662267
			Lebanon	PI428281, PI428286, PI428289, PI428291, PI428292, PI428294, PI428297, PI428298, PI428304, PI428307, PI428319, PI428320, PI428333, PI428334, PI428340, PI538734, PI538739, PI538740, PI538741, PI538743, PI538744, CItr17664, PI428261, PI428262, PI428263, PI428264, PI428265, PI428266, PI428267, PI428269, PI428281, PI428282, PI428283, PI428285, PI428288, PI428296, PI428300, PI428301, PI428302, PI428303, PI428314, PI428335
			Syria	PI487266, PI487267, PI487268, PI487270, PI662246, PI662250, PI662257, PI662258, PI662260, PI662270, PI662274, PI662251
			Turkey	PI428214, PI428222, PI428226, PI428254, PI538726
	30	-	Iran	PI662241
			Lebanon	PI428285, PI428308, PI428321, PI428325, PI428339, PI538745, PI538746, PI538747, PI662243, PI662254, PI662261, PI662279,

				PI428323, PI428327, PI428329, PI538742
			Syria	PI487269, PI662245, PI662276, PI662277
			Turkey	PI428213, PI428209, PI428223, PI428228, PI428233, PI428246, PI428318, PI538728
			Iraq	PI427328
S	63	-	Armenia	CItr17668, PI428181, PI428258, PI662244
			Iran	PI662240, PI662242
			Lebanon	PI428295, PI428305, PI538749, PI662280, PI428270, PI428324, PI538748
			Syria	PI487265, PI487271, PI662248, PI662253, PI662256, PI662268, PI662269, PI662271, PI662272, PI662275, PI662278, PI662281, PI662283
			Turkey	PI428184, PI428193, PI428198, PI428204, PI428207, PI428213, PI428220, PI428227, PI428229, PI428230, PI428231, PI428232, PI428234, PI428237, PI428238, PI428239, PI428240, PI428241, PI428242, PI428244, PI428245, PI428248, PI428249, PI428250, PI428251, PI428252, PI428255, PI538725, PI538731, PI538732, PI538733, PI554599
			Iraq	PI428316, PI662262
			-	CIer17669, CItr17669, D.V2138

Supplementary Table 5 ANK-NLRs, ANK-NLRs-WRKY, NLRs-WRKY proteins in grass genomes

Gene ID	Splice variants	CD-Search / hmmer	Species
TraesCS5D02G349800.1	TraesCS5D02G349800.2, TraesCS5D02G349800.3	NLR-WRKY	*Triticum aestivum*
TraesCS3D02G029100.1		NLR-WRKY	*Triticum aestivum*
TraesCS5A02G344100.1	TraesCS5A02G344100.2, TraesCS5A02G344100.3	ANK-NLR-WRKY	*Triticum aestivum*
TRIDC5AG050380		ANK-NLR-WRKY	*Triticum dicoccoides*
YrU1		ANK-NLR-WRKY	*Triticum urartu*
TRIUR3_13906		NLR-WRKY	*Triticum urartu*
TRIUR3_28246		NLR-WRKY	*Triticum urartu*
TRIUR3_01726		ANK-NLR	*Triticum urartu*
AET5Gv20793000.2	AET5Gv20793000.5, AET5Gv20793000.6	NLR-WRKY	*Aegilops tauschii* subsp. *strangulata*
AET3Gv20057800.1	AET3Gv20057800.2, AET3Gv20057800.3, AET3Gv20057800.4, AET3Gv20057800.7, AET3Gv20057800.8, AET3Gv20057800.9	NLR-WRKY	*Aegilops tauschii* subsp. *strangulata*
BRADI_4g09886v3.KQJ87226		NLR-WRKY	*Brachypodium distachyon*
OBART11G23060.1		NLR-WRKY	*Oryza barthii*
OBART11G22970.1	OBART11G22970.2	NLR-WRKY	*Oryza barthii*
OBART07G09910.1		NLR-WRKY	*Oryza barthii*
ONIVA03G01340.1	ONIVA03G01340.1，ONIVA03G01340.2	NLR-WRKY	*Oryza sativa f. spontanea*
OB11G27340.1		NLR-WRKY	*Oryza brachyantha*

**Gene ID**	**Splice variants**	**CD-Search / hmmer**	**Species**
ORUFI07G10000.1;		NLR-WRKY	*Oryza rufipogon*
BGIOSGA022298-PA		NLR-WRKY	*Oryza sativa* Indica Group
BGIOSGA035675-PA		NLR-WRKY	*Oryza sativa* Indica Group
BGIOSGA035677-PA		NLR-WRKY	*Oryza sativa* Indica Group
HORVU5Hr1G085900.8;	HORVU5Hr1G085900.1, HORVU5Hr1G085900.5; HORVU5Hr1G085900.10, HORVU5Hr1G085900.12, HORVU5Hr1G085900.13, HORVU5Hr1G085900.15	NLR-WRKY	*Hordeum vulgare* subsp. *vulgare*
-			*Zea mays*

Supplementary Table 6 ANK-containing proteins in *Triticum urartu* genome

Gene ID	CD-Search / hmmer	Gene ID	CD-Search / hmmer
TRIUR3_01726	Ank_2-NLR	TRIUR3_19319	Ank_2-Ank_3-Ank_2
TRIUR3_16022	Ank_4	TRIUR3_15310	Ank_2-Ank_4
TRIUR3_15383	PPR- PPR_2- PPR- PPR- PPR- PPR- PPR_2- Ank_2-PGG	TRIUR3_22857	Ank_2-Ank_4
TRIUR3_02015	Ank- Ank_2- TPR_1	TRIUR3_25013	Ank_2-Ank_4-Ank-PGG
TRIUR3_27595	Ank_2	TRIUR3_26705	Ank_2-Ank_4-PGG
TRIUR3_33180	Ank_2	TRIUR3_16218	Ank_2-Ank_4-PGG
TRIUR3_29874	Ank_2	TRIUR3_16330	Ank_2-Ank_5-PGG
TRIUR3_20538	Ank_2	TRIUR3_19983	Ank_2-Ank_5-PGG
TRIUR3_06394	Ank_2- Ank_4- TPR_2	TRIUR3_01361	Ank_2-Ank_5-PGG
TRIUR3_24498	Ank_2-Ank_2	TRIUR3_21746	Ank_2-Ank-PGG
TRIUR3_16658	Ank_2-Ank_2	YrU1	Ank_2-NLR-WRKY
TRIUR3_29116	Ank_2-Ank_2	TRIUR3_15871	Ank_2-PGG
TRIUR3_30670	Ank_2-Ank_2-Ank	TRIUR3_21880	Ank_2-PGG
TRIUR3_20368	Ank_2-Ank_2-Ank_2	TRIUR3_15982	Ank_4-Ank_2
TRIUR3_35360	Ank_2-Ank_2-Ank_2-PGG	TRIUR3_15002	Ank_4-Ank_2-PGG
TRIUR3_11541	Ank_2-Ank_2-Ank_2-PGG	TRIUR3_17320	Ank_4-Ank_2-PGG
TRIUR3_31069	Ank_2-Ank_2-Ank_2-TPR_2	TRIUR3_12922	Ank_5-PGG
TRIUR3_34062	Ank_2-Ank_2-Ank_4	TRIUR3_22856	Ank_5-PGG
TRIUR3_03569	Ank_2-Ank_2-Ank_4-Ank_2-PGG	TRIUR3_27218	Ank_5-PGG
TRIUR3_29915	Ank_2-Ank_2-Ank_4-PGG	TRIUR3_00453	Ank_5-PGG
TRIUR3_30034	Ank_2-Ank_2-Ank-PGG	TRIUR3_00457	Ank_5-PGG
TRIUR3_33157	Ank_2-Ank_2-Ank-TPR_1	TRIUR3_03294	Ank-Ank_2
TRIUR3_24244	Ank_2-Ank_2-PGG	TRIUR3_12904	Ank-Ank_5
TRIUR3_10603	Ank_2-Ank_2-PGG	TRIUR3_12440	Ank-Ank_5-PGG
TRIUR3_11540	Ank_2-Ank_2-PGG	TRIUR3_25229	NB-ARC- BTB-DUF3420- Ank_2-NPR1_like_C
TRIUR3_05606	Ank_2-Ank_2-PGG	TRIUR3_16250	Pkinase-Ank_2-Ank_2-Ank_2
TRIUR3_15309	Ank_2-Ank_2-PGG	TRIUR3_31911	Spindle_Spc25-Ank_2-Ank_2-PGG

Supplementary Table 7 WRKY-containing proteins in *Triticum urartu* genome

Gene ID	CD-Search / hmmer	Gene ID	CD-Search / hmmer
YrU1	ANK-NLR-WRKY	TRIUR3_03043	WRKY
TRIUR3_28246	NLR-WRKY	TRIUR3_23877	WRKY
TRIUR3_13906	NLR-WRKY	TRIUR3_29978	WRKY
TRIUR3_21369	Plant_zn_clust-WRKY	TRIUR3_16804	WRKY
TRIUR3_28409	Plant_zn_clust-WRKY	TRIUR3_22129	WRKY
TRIUR3_33054	Plant_zn_clust-WRKY	TRIUR3_16097	WRKY
TRIUR3_20596	Plant_zn_clust-WRKY	TRIUR3_33327	WRKY
TRIUR3_09583	ULP1-WRKY	TRIUR3_24639	WRKY
TRIUR3_29889	WRKY	TRIUR3_34281	WRKY
TRIUR3_30416	WRKY	TRIUR3_25260	WRKY
TRIUR3_05298	WRKY	TRIUR3_31234	WRKY
TRIUR3_24602	WRKY	TRIUR3_22321	WRKY
TRIUR3_12546	WRKY	TRIUR3_24321	WRKY
TRIUR3_22128	WRKY	TRIUR3_33662	WRKY
TRIUR3_17401	WRKY	TRIUR3_06407	WRKY
TRIUR3_04352	WRKY	TRIUR3_01447	WRKY
TRIUR3_27575	WRKY	TRIUR3_13752	WRKY
TRIUR3_20905	WRKY	TRIUR3_01445	WRKY
TRIUR3_16697	WRKY	TRIUR3_26868	WRKY
TRIUR3_25300	WRKY	TRIUR3_31797	WRKY
TRIUR3_06813	WRKY	TRIUR3_14306	WRKY
TRIUR3_21738	WRKY	TRIUR3_27037	WRKY
TRIUR3_06231	WRKY	TRIUR3_17960	WRKY
TRIUR3_03056	WRKY	TRIUR3_28158	WRKY
TRIUR3_23069	WRKY	TRIUR3_10995	WRKY
TRIUR3_26571	WRKY	TRIUR3_26595	WRKY
TRIUR3_22178	WRKY	TRIUR3_27149	WRKY
TRIUR3_34396	WRKY	TRIUR3_21018	WRKY-WRKY
TRIUR3_03769	WRKY	TRIUR3_22206	WRKY-WRKY
TRIUR3_23179	WRKY	TRIUR3_31607	WRKY-WRKY
TRIUR3_07596	WRKY	TRIUR3_02376	WRKY-WRKY
TRIUR3_10262	WRKY	TRIUR3_09883	WRKY-WRKY
TRIUR3_16803	WRKY	TRIUR3_24481	WRKY-WRKY

TRIUR3_31959	WRKY	TRIUR3_20325	WRKY-WRKY
TRIUR3_16802	WRKY	TRIUR3_28514	WRKY-WRKY
TRIUR3_35037	WRKY	TRIUR3_09462	WRKY-WRKY
TRIUR3_10994	WRKY	TRIUR3_27317	WRKY-WRKY
TRIUR3_01979	WRKY	TRIUR3_17462	WRKY-WRKY-WRKY

Supplementary Table 8 Primers used in this study

Primer name	Forward(5'-3')	Reverse(5'-3')
**For cloning and complementation**		
YrU1-C	ATGGAGCAGCAGCAGAGCTTCGAGG	TTACGTGCTAACATTATCAAGTGGA
CG2-C	AAGGAGGTGCGCGAGCTGTC	TGACAAGTGCTCGATGCCAA
YrU1-XbaI/PstI	CCGGGGATCCTCTAGAGTGATGTTTGATAGCACACAAGGTA	CCAAGCTTGCATGCCTGCAGATGCCATCCTCTCTACTCGGTCTTT
YrU1 N-tag	GGGGACAAGTTTGTACAAAAAAGCAGGCTTCATGGAGCAGCAGCAGAGCTTCGAGG	GGGGACCACTTTGTACAAGAAAGCTGGGTCTCAAACTCGGTCAAATTCGTAGCCA
YrU1-HA	TAGAACTAGTGGATCCATGGAGCAGCAGCAGAGCTTCGAGG	CCCCCTCGAGGTCGACAACTCGGTCAAATTCGTAGCCATCG
YrU1-ANK-HA	TAGAACTAGTGGATCCATGGAGCAGCAGCAGAGCTTCGAGG	CCCCCTCGAGGTCGACCATAGCACGGTCTTCCGCTCTCCCT
YrU1-CC-HA	TAGAACTAGTGGATCCATGAGTAGTCAAAAGCTCCACACTG	CCCCCTCGAGGTCGACATCCTTACCCAACCACGCTTCA
YrU1-NB-ARC-HA	TAGAACTAGTGGATCCATGCATTTGAATACTCTTGAAGCGT	CCCCCTCGAGGTCGACTCGACGAACCTTGTCTGCGAGT
YrU1-LRR-HA	TAGAACTAGTGGATCCATGGATGATTTGGTAAAGCAATGGA	CCCCCTCGAGGTCGACCATCCTCCGACTACTCATCTTCCT
YrU1-WRKY-HA	TAGAACTAGTGGATCCATGCCCAGGCGGACCAGGAAG	CCCCCTCGAGGTCGACAACTCGGTCAAATTCGTAGCCATCG
YrU1-ANK-MYC/GFP	GGGGCCCGGGGTCGACATGGAGCAGCAGCAGAGCTTCGAGG	TACCGGATCCACTAGTCATAGCACGGTCTTCCGCTCTCCCT
YrU1-CC-MYC/GFP	GGGGCCCGGGGTCGACATGAGTAGTCAAAAGCTCCACACTG	TACCGGATCCACTAGTATCCTTACCCAACCACGCTTCA
YrU1-ANK N-tag	GGGGACAAGTTTGTACAAAAAAGCAGGCTTCATGGAGCAGCAGCAGAGCTTCGAGG	GGGGACCACTTTGTACAAGAAAGCTGGGTCTCACATAGCACGGTCTTCCGCTCTCCCT
YrU1-CC N-tag	GGGGACAAGTTTGTACAAAAAAGCAGGCTTCATGAGTAGTCAAAAGCTCCACACTGAGG	GGGGACCACTTTGTACAAGAAAGCTGGGTCTCAATCCTTACCCAACCACGCTTCA
YrU1-NB-ARC N-tag	GGGGACAAGTTTGTACAAAAAAGCAGGCTTCATGCATTTGAATACTCTTGAAGCGT	GGGGACCACTTTGTACAAGAAAGCTGGGTCTCATCGACGAACCTTGTCTGCGAGT
YrU1-LRR N-tag	GGGGACAAGTTTGTACAAAAAAGCAGGCTTCATGGATGATTTGGTAAAGCAATGGA	GGGGACCACTTTGTACAAGAAAGCTGGGTCTCACATCCTCCGACTACTCATCTTCCT
YrU1-WRKY N-tag	GGGGACAAGTTTGTACAAAAAAGCAGGCTTCATGCCCAGGCGGACCAGGAAGATGAGTA	GGGGACCACTTTGTACAAGAAAGCTGGGTCTCAAACTCGGTCAAATTCGTAGCCATCG
**For 3’ and 5’ RACE**		
YrU1-3’	TCCCACCGCCAGCATACAGTGTTTAGAT	
YrU1-5’	ACCTCCTTGGTCAGGCACTTGCTCGTC	

**For map-based cloning**		
Xgwm186-5A	GCAGAGCCTGGTTCAAAAAG	CGCCTCTAGCGAGAGCTATG
SCF1	GAAACCAGCAAAGAAAGAAA	CGTAGCAGTCATGCAATTTA
SCF2	GTTGCTGCAAACAACAATAA	ATGTAGATCGCAACCCATAG
SCF3	CTAGAACCTTTTCAGAACCG	GAAAAAGAAAAATGTTCACGA
SCF4	AAAACAAATAATGGTGACCG	AGCCAGAAATATGCTGATGT
SCF5	ATGTTTGTAACCGACTCTGG	TTTTCACTAGCAAAAGGGTC
SCF6	CAACCACCCAGTATTGTTCT	AGCTGCAGACTTTTAACGAC
SCF19	GTTTGTAGCAGACAACAACAAC	ACCAAAGAGTTAGCTATGGACA
SCF20	GGATCTCAGACATGTACGCT	CATTACTAGGTACCGGCTTG
SCF21	AACTGCTCAAGAGCTGTAGG	AGGAACCTGCTTACAAATCA
SCF22	TCTTTGTTCACAAATCACGA	AATATGACCAAGCCTGAGTG
SCF10	GTAGGAGTACCACGCAACAT	GTAATTTGTTTATCCACCGC
SCF11	ATGTGTATGTGTGAATGGTTGT	GTTTTAGGATTTTAGGGGTTTT
SCF12	TGCTGTGTTGCTGAGATAAG	TATGTTACACCCACCGGTAT
SCF13	GCTGCTCCTGCTCTAGTAAA	AAGTAATAAGCGCACCAAAC
Xgpw7007-5A	AGGAGAGCGGCAAGGACTAC	GAGAATCACAGGAAGGCACG
SCF14	AATCCATCGATAGCAAGAGA	TCATTCATCCAAAACATCAA
SCF15	GGTCAATCAACGACCAGTAT	ACACAAGAAGAAAACGGAAA
SCF16	GTTCTGCATTACCTGGAAAG	CCAATTATGGTTGGTGTTCT
SCF17	TCGATGGTCAAACCTAAAAT	ACACTTTTGCAGGTTGAAAT
SCF18	CACCTGTTGAGAGGAAGAAG	AGAATGCCAAACTGATTCAT
SCF23	AGAACCATCCGAATAGGCCGAGAC	AGAGGTGGATTGGGCATGGAAAC
SCF24	CGGTGGTGGTGAAGATTTGAA	GACGAACCTTGTCTGCGAGTG
SCF25	GGCAAAGCAATGGATAGCCGAAGG	TGCAACAAGCTGGGCAAATGAAC
SCF26	CGAGGACACCAACCAGATAGC	CCAGGCAGAAAGACAGGAAAA
CHL14	TTGGATGGGCATTTTTCCGC	TCCAAGATACAAGTGTCCGGC
3722	GTCCCTTGCCAACCTGTCAGGATAG	CTGCATGCCATTCTTCAGAAGCTAA
Xcfa2155-5A	TTTGTTACAACCCAGGGGG	TTGTGTGGCGAAAGAAACAG
Xgwm595-5A	GCATAGCATCGCATATGCAT	GCCACGCTTGGACAAGATAT
YrU1-M	AGAGTGCACCATCCGTGCCGGGTTG	ACTCCCGCTTCCGCTTACCACCTCG
**For validation and test**		
YrU1-1738	TGGGAAGATGCTCACAGAAGGTA	CTCAAACTCGGTCAAATTCGTAG
**For VIGS**		
YrU1-VIGS	TTTTTTTTTTTTTTTAGCTAGCGCACACTGCATCACATGGTA	GATTCTTCTTCCGTTGCTAGCACTTGAAGAAGCCGAAACTC
**For real-time PCR**		

Actin	AAACCTTCAGTTGCCCAG	CTCACACCATCACCAGAGTC
YrU1-1	TGCTCTTGTATTTCTGTGTCTCTGT	CGTCAAAAAGGCACGTGGGAAGATG
YrU1-2	GCTACTGAAGCCCGATGTCTC	TCTCCAGCTCCATTGATTGC
TuPR1	CAGGACTACGACTATGGCTC	GTCCAATGATATTCCCGCGG
TuPR2	GTTCGCTGCTGTTCCTATGAGTGT	CGTTGATGCCCTTGGACCTGTAG
TuPR3	AGGATGTTGCTTCCATGTTTGCCG	AAGTAGATGCGCATGCCGTTGATG
TuPR5	GCAGCACCCAGGACTTCTACGA	GCGTGTGTGGCTTGGTCGTT
**For Luciferase complementation imaging assay**		
YrU1-nLUC	TCGGTACCCGGGATCCAATGGAGCAGCAGCAGAGCTTCGAGG	ACGAGATCTGGTCGACAACTCGGTCAAATTCGTAGCCA
YrU1-ANK-nLUC	TCGGTACCCGGGATCCAATGGAGCAGCAGCAGAGCTTCGAGG	ACGAGATCTGGTCGACCATAGCACGGTCTTCCGCTCTCCCT
YrU1-CC-nLUC	TCGGTACCCGGGATCCAAGTAGTCAAAAGCTCCACACTGAGG	ACGAGATCTGGTCGACATCCTTACCCAACCACGCTTCA
YrU1-ANK-cLUC	GCGGTACCCGGGATCCAATGGAGCAGCAGCAGAGCTTCGAGG	TACGAACGAAAGCTCTGCAGTCACATAGCACGGTCTTCCGCTCTCCCT
YrU1-CC-cLUC	GCGGTACCCGGGATCCAAGTAGTCAAAAGCTCCACACTGAGG	TACGAACGAAAGCTCTGCAGTCAATCCTTACCCAACCACGCTTCA
YrU1-cLUC	GCGGTACCCGGGATCCAATGGAGCAGCAGCAGAGCTTCGAGG	TACGAACGAAAGCTCTGCAGTCAAACTCGGTCAAATTCGTAGCCA
**For Yeast two-hybrid analysis**		
YrU1-BD	AGGAGGACCTGCATATGATGGAGCAGCAGCAGAGCTTCGAGG	GGATCCCCGGGAATTCTCAAACTCGGTCAAATTCGTAGCCA
YrU1-ANK-BD	AGGAGGACCTGCATATGAGCAGCAGAGCTTCGAGG	GGATCCCCGGGAATTCTCACATAGCACGGTCTTCCGCTCTCCCT
YrU1-CC-BD	AGGAGGACCTGCATATGAGTAGTCAAAAGCTCCACACTGAGG	GGATCCCCGGGAATTCTCAATCCTTACCCAACCACGCTTCA
YrU1-AD	CAGATTACGCTCATATGATGGAGCAGCAGCAGAGCTTCGAGG	CACCCGGGTGGAATTCTCAAACTCGGTCAAATTCGTAGCCA
YrU1-ANK-AD	CAGATTACGCTCATATGATGGAGCAGCAGCAGAGCTTCGAGG	CACCCGGGTGGAATTCTCACATAGCACGGTCTTCCGCTCTCCCT
YrU1-CC-AD	CAGATTACGCTCATATGAGTAGTCAAAAGCTCCACACTGAGG	CACCCGGGTGGAATTCTCAATCCTTACCCAACCACGCTTCA
