# Supplementary material for: An ankyrin-repeat and WRKY-domain-containing immune receptor confers stripe rust resistance in wheat
Source: Nat Commun. 2020 Mar 13;11:1353. doi: 10.1038/s41467-020-15139-6 (PMC7070047; doi:10.1038/s41467-020-15139-6)
Supplement: Supplementary file 3 — Reporting Summary [file 41467_2020_15139_MOESM3_ESM.pdf]

nature research

Corresponding author(s): Dingzhong Tang

Last updated by author(s): Feb 7, 2020

Reporting Summary

Nature Research wishes to improve the reproducibility of the work that we publish. This form provides structure for consistency and transparency in reporting. For further information on Nature Research policies, seeAuthors & Referees and theEditorial Policy Checklist .

Statistics

For all statistical analyses, confirm that the following items are present in the figure legend, table legend, main text, or Methods section.

n/a	Confirmed	
☐	☒	The exact sample size (*n*) for each experimental group/condition, given as a discrete number and unit of measurement
☐	☒	A statement on whether measurements were taken from distinct samples or whether the same sample was measured repeatedly
☐	☒	The statistical test(s) used AND whether they are one- or two-sided<br>*Only common tests should be described solely by name; describe more complex techniques in the Methods section.*
☒	☐	A description of all covariates tested
☒	☐	A description of any assumptions or corrections, such as tests of normality and adjustment for multiple comparisons
☒	☐	A full description of the statistical parameters including central tendency (e.g. means) or other basic estimates (e.g. regression coefficient) AND variation (e.g. standard deviation) or associated estimates of uncertainty (e.g. confidence intervals)
☐	☒	For null hypothesis testing, the test statistic (e.g. *F*, *t*, *r*) with confidence intervals, effect sizes, degrees of freedom and *P* value noted<br>*Give P values as exact values whenever suitable.*
☒	☐	For Bayesian analysis, information on the choice of priors and Markov chain Monte Carlo settings
☒	☐	For hierarchical and complex designs, identification of the appropriate level for tests and full reporting of outcomes
☒	☐	Estimates of effect sizes (e.g. Cohen's *d*, Pearson's *r*), indicating how they were calculated

Our web collection on statistics for biologists contains articles on many of the points above.

Software and code

Policy information about availability of computer code

Data collection	No software was used for data collection.
Data analysis	SSR Locator software: tool to designed the SSR markers in Map-based cloneing.<br>Softberry: predicted of coding sequence(http://www.softberry.com/berry.phtml?topic=fgenesh&group=programs&subgroup=gfind).<br>DNAMAN(v9.0.1.116): translated of coding sequence.<br>SMART: prediction of conserved domain(http://smart.embl-heidelberg.de/).<br>LRRsearch: prediction of leucine-rich repeats (LRRs)(http://lrrsearch.com/).<br>CD-search: conserved domain prediction program(https://www.ncbi.nlm.nih.gov/Structure/cdd/wrpsb.cgi).<br>MEGA(v7.0.26): phylogenetic analyses.<br>Hmmer (v3.1b2): searching sequence databases for sequence homologs (http://hmmer.org/).<br>PASW Statistcs 18: data statistic analysis.<br>ClustalX(v2.1): program of sequence alignment analysis.<br>JionMap(v4.0): genetic map construction.

For manuscripts utilizing custom algorithms or software that are central to the research but not yet described in published literature, software must be made available to editors/reviewers. We strongly encourage code deposition in a community repository (e.g. GitHub). See the Nature Research guidelines for submitting code & software for further information.

Data

Policy information about availability of data

All manuscripts must include a data availability statement. This statement should provide the following information, where applicable:

- Accession codes, unique identifiers, or web links for publicly available datasets
- A list of figures that have associated raw data
- A description of any restrictions on data availability

Data that supporting the findings of this study are presented in the manuscript and the supplementary files. GenBank accession of YrU1 gene in PI428309 is MT018453. Source data of Fig. 1, 3, 4 and 6, as well as Supplementary Figs. 1-4, 7-9 and 11 are provided as a Source Data file.

Field-specific reporting

Please select the one below that is the best fit for your research. If you are not sure, read the appropriate sections before making your selection.

[x] Life sciences [ ] Behavioural & social sciences [ ] Ecological, evolutionary & environmental sciences

For a reference copy of the document with all sections, see nature.com/documents/nr-reporting-summary-flat.pdf

Life sciences study design

All studies must disclose on these points even when the disclosure is negative.

Sample size	The sample size were listed in the specific experiments.
Data exclusions	No data were excluded from the analyses.
Replication	All experiments were repeated at least two to three times.
Randomization	The phenotypes of rust resistance and RT-PCR experiments were performed using randomization.
Blinding	Blinding was preformed when the rust inoculation tests.

Reporting for specific materials, systems and methods

We require information from authors about some types of materials, experimental systems and methods used in many studies. Here, indicate whether each material, system or method listed is relevant to your study. If you are not sure if a list item applies to your research, read the appropriate section before selecting a response.

Materials & experimental systems

n/a	Involved in the study	
[ ]	[x]	Antibodies
[x]	[ ]	Eukaryotic cell lines
[x]	[ ]	Palaeontology
[x]	[ ]	Animals and other organisms
[x]	[ ]	Human research participants
[x]	[ ]	Clinical data

Methods

n/a	Involved in the study	
[x]	[ ]	ChIP-seq
[x]	[ ]	Flow cytometry
[x]	[ ]	MRI-based neuroimaging

Antibodies

Antibodies used	Commercial antibodies: α-GFP (7G9, M2004, Abmart, 1:2000 dilution), α-MYC(192C, M2002, Abmart, 1:5000 dilution), α-HA (26D11, M2003, Abmart, 1:1000 dilution), HRP, Goat Anti-Mouse IgG (A21010, Abbkine, 1:10000 dilution), Anti-GFP (Green Fluorescent Protein) mAb-Agarose(D153-8, MBL).
Validation	The validation of the commercial antibodies are provided with the manufacturers: α-GFP (http://www.ab-mart.com.cn/upload/20170614093556xz.pdf), α-MYC (http://www.ab-mart.com.cn/upload/20170614093526xz.pdf), α-HA (http://www.ab-mart.com.cn/upload/20170614093540xz.pdf). HRP, Goat Anti-Mouse IgG (http://www.abbkine.cn/product/A21010).
