# Supplementary material for: An ankyrin-repeat and WRKY-domain-containing immune receptor confers stripe rust resistance in wheat
Source: Nat Commun. 2020 Mar 13;11:1353. doi: 10.1038/s41467-020-15139-6 (PMC7070047; doi:10.1038/s41467-020-15139-6)
Supplement: Supplementary file 4 — Description of Additional Supplementary Files [file 41467_2020_15139_MOESM4_ESM.pdf]

Description of Additional Supplementary Files

File Name: Supplementary Data 1
Description: Sequence data of the genomic sequence, the coding sequence and predicted protein sequence of the *YrU1* gene in PI428309 are shown. YrU1 locus+UTRs: the 11,858-bp genomic fragment of *YrU1*, including the 2,714 bp upstream of the start codon, all the exons and introns and 2,086 bp downstream of the stop codon. YrU1 CDS: the coding sequence of the *YrU1* gene. YrU1 protein: the amino acid sequence of YrU1. The *YrU1* gene encodes an ANK-NLR-WRKY protein.
